# A New Insight into the Physiological Role of Bile Salt Hydrolase among Intestinal Bacteria from the Genus *Bifidobacterium*

**DOI:** 10.1371/journal.pone.0114379

**Published:** 2014-12-03

**Authors:** Piotr Jarocki, Marcin Podleśny, Paweł Glibowski, Zdzisław Targoński

**Affiliations:** 1 Department of Biotechnology, Human Nutrition and Food Commodities, University of Life Science in Lublin, Lublin, Poland; 2 Department of Milk Technology and Hydrocolloids, University of Life Science in Lublin, Lublin, Poland; Charité, Campus Benjamin Franklin, Germany

## Abstract

This study analyzes the occurrence of bile salt hydrolase in fourteen strains belonging to the genus *Bifidobacterium*. Deconjugation activity was detected using a plate test, two-step enzymatic reaction and activity staining on a native polyacrylamide gel. Subsequently, bile salt hydrolases from *B. pseudocatenulatum* and *B. longum* subsp. *suis* were purified using a two-step chromatographic procedure. Biochemical characterization of the bile salt hydrolases showed that the purified enzymes hydrolyzed all of the six major human bile salts under the pH and temperature conditions commonly found in the human gastrointestinal tract. Next, the dynamic rheometry was applied to monitor the gelation process of deoxycholic acid under different conditions. The results showed that bile acids displayed aqueous media gelating properties. Finally, gel-forming abilities of bifidobacteria exhibiting bile salt hydrolase activity were analyzed. Our investigations have demonstrated that the release of deconjugated bile acids led to the gelation phenomenon of the enzymatic reaction solution containing purified BSH. The presented results suggest that bile salt hydrolase activity commonly found among intestinal microbiota increases hydrogel-forming abilities of certain bile salts. To our knowledge, this is the first report showing that bile salt hydrolase activity among *Bifidobacterium* is directly connected with the gelation process of bile salts. In our opinion, if such a phenomenon occurs in physiological conditions of human gut, it may improve bacterial ability to colonize the gastrointestinal tract and their survival in this specific ecological niche.

## Introduction

So far, bile salt hydrolase (BSH) activity has been detected in many bacterial species living in the gastrointestinal tract of human and animals [1], [2]. This enzyme which catalyzes hydrolysis of conjugated bile salts into amino acid residues and bile acids is particularly evident among bifidobacteria, which are an important part of normal human bacterial flora commonly found in the intestine. Most of the examined bifidobacterial species possess BSH activity and, interestingly, their activity is much higher than that of other microbial inhabitants of the human gut e.g. from the genus *Lactobacillus* [3], [4]. Although this enzymatic activity is very common, only a few *Bifidobacterium* BSHs have been thoroughly analyzed [5]–[8]. Additionally, the importance of this enzyme for the bacterial cells is also not well understood. Although several hypotheses have been proposed, the precise physiological function of bile salt hydrolases currently remains unknown. It has been hypothesized that the conjugation of bile salts may be a mechanism of bile detoxication, and therefore, these enzymes may play an important role in microbial bile tolerance and survival in the intestine [9]. Some reports in literature show, however, that the unconjugated bile acids have a greater inhibitory effect on bacteria than their conjugated forms, hence direct correlation of BSH activity with bile tolerance is not evident [10]. Other researchers suggest that amino acids liberated from bile salt could be used as carbon, nitrogen and even energy sources for bacteria [5], [11]. On the other hand, Tannock et al. [10] and Gilliland and Speck [12] proved that the BSH-positive lactobacilli do not utilize the steroid moiety of the bile salts. Moreover, taurine contains a sulfonic moiety that is dissimilated to hydrogen sulfide which can be toxic to both bacteria and human host [2], [13]. Finally, it is possible that BSH facilitates incorporation of cholesterol moiety into bacterial membranes. This phenomenon may change fluidity, permeability and net charge of bacterial membranes and thereby improve the colonization ability and survival of these microbes in the gut [14]–[16]. However, although some research showed an essential role of BSH in the persistence of bacteria in the intestines [40], [41], the knowledge about the importance of BSH activity for bifidobacteria is still limited, and therefore further studies in this direction are necessary.

Previous reports have suggested the feasibility of using lactic acid bacteria to reduce serum cholesterol concentration [15], [17]. These cholesterol-lowering effects can be ascribed to different mechanisms, such as assimilation of cholesterol by the bacteria, binding of cholesterol to the bacterial cell walls and deconjugation of bile salts in the presence of bile salt hydrolases [17]–[19]. Because deconjugated bile salts are less water-soluble, they are also less efficiently reabsorbed compared with their conjugated forms. This phenomenon results in the enhanced excretion of free bile acids in feces leading to the increased requirement for cholesterol, which is a precursor for the synthesis of bile salts. Therefore, a high BSH activity in the intestine could finally lead to a reduction in serum cholesterol [20]–[22].

On the other hand, some researchers suggest adverse effects of BSH activity on human health. It has been postulated that the deconjugation of bile salts in the intestine may impair normal lipid digestion and colonic mucosal function, have a role in gallstone formation and even promote colon cancer [23]–[25]. These effects seem to be especially important as some strains from the genus *Lactobacillus* and *Bifidobacterium* displaying BSH activity are commonly used as probiotics in the dairy industry.

In this study, we analyzed the occurrence of bile salt hydrolase in fourteen strains belonging to the *Bifidobacterium* genus. Two BSHs from *B. pseudocatenulatum* and *B. longum* subsp. *suis* were purified and analyzed for their selected biochemical and molecular features. Finally, hydrogel-forming abilities of BSH-positive bifidobacteria were studied in the presence of different bile salts.

## Materials and Methods

### Microorganisms, media and culture conditions

All bacterial strains used in this study (Table 1) were obtained from the German Collection of Microorganisms (DSMZ) and ARS culture collection (NRRL). Bifidobacteria were cultured in a modified Garche's medium (peptone, 20 g/l; yeast extract, 2 g/l; lactose, 10 g/l; L-cysteine hydrochloride, 0.4 g/l; sodium acetate, 6 g/l; MgSO_4_×7H_2_O, 0.12 g/l; KH_2_PO_4_, 2 g/l; Na_2_HPO_4_×12 H_2_O 2.5 g/l; pH 6.4) [26] or in a standard MRS medium supplemented with L-cysteine hydrochloride (0.4 g/l). To detect bile salt hydrolase activity, media were solidified with 1.5% of agar and, additionally, 0.25% (w/v) of sodium salt of taurodeoxycholic acid was added. Hydrogel-forming abilities of bifidobacteria were observed using Garche's medium plates that contained different concentrations (0 – 1%) of taurocholate, taurodeoxycholate and glycochenodeoxycholate. Cultures and plates were incubated anaerobically at 37°C using anaerobic jars and AnaeroGen sachets (Oxoid).

**Table 1:** List of bacterial strains used in this study.

Strain	Source of isolation	BSH activity
*Bifidobacterium adolescentis* DSM 20087	bovine rumen	+
*Bifidobacterium animalis* subps. *animalis* NRRL B-41406^ T^	rat feces	*+*
*Bifidobacterium animalis* subsp. *lactis* NRRL B-41405	yoghurt	*+*
*Bifidobacterium asteroides* DSM 20089^ T^	hindgut of honeybee	−
*Bifidobacterium bifidum* DSM 20456^ T^	stool of breast-fed infant	+
*Bifidobacterium breve* DSM 20091	intestine of infant	+
*Bifidobacterium breve* NRRL B-41408^ T^	intestine of infant	+
*Bifidobacterium catenulatum* DSM 20224	sewage	+
*Bifidobacterium coryneforme* DSM 20216^ T^	hindgut of honeybee	−
*Bifidobacterium longum* subsp. *infantis* ATCC 15697^ T^	intestine of infant	+
*Bifidobacterium longum* subsp. *longum* NRRL B-41409^ T^	intestine of adult	*+*
*Bifidobacterium longum* subsp. *suis* NRRL B-41407^ T^	pig feces	+
*Bifidobacterium pseudocatenulatum* DSM 20439	sewage	+
*Bifidobacterium pseudolongum* subsp. *pseudolongum* DSM 20095	Chicken faeces	+
*Lactobacillus rhamnosus* NRRL B-442^ T^	-	−

### Cell extracts preparation and BSH assays

For cell-free extracts preparation, bifidobacteria were grown in 500 ml of a Garche's medium or MRS broth for 24 h, and then cells were harvested by centrifugation at 10,000 ×g for 10 min at 4°C. The cell pellet was washed twice in a 0.1 M sodium-phosphate buffer (pH 7.0) and resuspended in 20 ml of the same buffer containing 10 mM 2-mercaptoethanol (Sigma Aldrich). The cell suspensions were disrupted by sonication for 3 min with constant cooling, followed by centrifugation at 20,000 ×g for 10 min at 4°C. The obtained supernatants were stored at −20°C. Next, BSH activity was measured by determining the amount of liberated amino acids from the conjugated bile salts. Briefly, to 190 µl of a 0.1 M sodium-phosphate buffer (pH 6.0) containing 2-mercaptoethanol and of conjugated bile salt (both at final concentration of 10 mM), 10 µl of appropriately diluted protein sample was added. The mixture was incubated at 37°C for 30 min and then reaction was terminated by adding 200 µl of 15% (w/v) TCA. To remove the precipitate, samples were centrifuged at 14,000 ×g. Next, the amounts of released amino acids were measured by the ninhydrin assay as described previously [4]. One unit of BSH activity was defined as the amount of enzyme that can liberate 1 µmol of amino acid from a substrate per minute. Protein concentrations were measured by Bradford method [48], and bovine serum albumin was used as a standard.

### Native PAGE and BSH activity staining

Native electrophoresis was performed on Mini Protean II system (Bio-Rad) using a nondenaturating 10% (wt/vol) acrylamide gel with Laemmli buffer system omitting SDS [27]. For most of the strains, about 50 µg of protein sample was loaded on gel. In the case of *B. animalis* subsp. *lactis* and *B. longum* subsp. *suis*, 10 µg and 30 µg of protein sample were used, respectively. Immediately after electrophoretic separation, the gel was washed twice in a 0.4 M sodium-acetate buffer (pH 4.5) with 10 mM 2-mercaptoethanol and then incubated at 37°C for 1 h in a 0.1 M sodium-phosphate buffer (pH 6.0) containing 10 mM 2-mercaptoethanol and 10 mM sodium salt of taurodeoxycholic acid or glycodeoxycholic acid. The BSH activity in the gel appeared as a white precipitate of deoxycholic acid at the position of the enzyme.

### Proteomic analyses – MS and protein identification

Protein samples were excised from the gels and analyzed by liquid chromatography coupled with the mass spectrometry at the Laboratory of Mass Spectrometry, Institute of Biochemistry and Biophysics, Polish Academy of Sciences (Warsaw, Poland). Samples were concentrated and desalted on a RP-C18 pre-column (Waters), and peptides were separated on a nano-Ultra Performance Liquid Chromatography (UPLC) RP-C18 column (Waters, BEH130 C18 column, 75 µm i.d., 250 mm long) of a nanoACQUITY UPLC system, using a 45-min linear acetonitrile gradient. Column outlet was directly coupled to the Electrospray ionization (ESI) ion source of the Orbitrap Velos type mass spectrometer (Thermo), working in the regime of data dependent MS to MS/MS switch with HCD type peptide fragmentation. An electrospray voltage of 1.5 kV was used. Raw data files were pre-processed with Mascot Distiller software (version 2.4.2.0, MatrixScience). The obtained peptide masses and fragmentation spectra were matched to the National Center Biotechnology Information (NCBI) non-redundant database (31002772 sequences/10668937692 residues), with a Bacteria filter (20,812,975 sequences) using the Mascot search engine (Mascot Daemon v. 2.4.0, Mascot Server v. 2.4.1, MatrixScience). The following search parameters were applied: enzyme specificity was set to trypsin, peptide mass tolerance to ± 30 ppm and fragment mass tolerance to ± 0.1 Da. The protein mass was left as unrestricted, and mass values as monoisotopic with one missed cleavage being allowed. Alkylation of cysteine by carbamidomethylation as fixed, and oxidation of methionine was set as a variable modification. Protein identification (Table 2) was performed using the Mascot search engine (MatrixScience), with the probability-based algorithm. The expected value threshold of 0.05 was used for analysis, which means that all peptide identifications had less than 1 in 20 chance of being a random match.

**Table 2:** LC-MS/MS analysis of protein samples obtained from native PAGE.

Sample	Gi number	Protein name	Protein score^a^ (peptide no^b^)	Sequence coverage [%]
1	gi|489905722	choloylglycine hydrolase [*B. adolescentis*]	743 (8)	36
2	gi|386867035	bile salt hydrolase [*B. animalis* subsp. *animalis* ATCC 25527]	780 (9)	41
3	gi|183219214	bile salt hydrolase [*B. animalis* subsp. *lactis* HN019]	379 (6)	27
4.1	gi|489913385	choloylglycine hydrolase [*B. bifidum*]	582 (6)	30
4.2	gi|489913385	choloylglycine hydrolase [*B. bifidum*]	888 (9)	43
5	gi|23465372	choloylglycine hydrolase [*B. longum* NCC2705]	899 (9)	44
6	gi|23465372	choloylglycine hydrolase [*B. longum* NCC2705]	765 (8)	39
7	gi|489931556	choloylglycine hydrolase [*B. catenulatum*]	925 (9)	43
8	gi|213692328	choloylglycine hydrolase [*B. longum* subsp. *infantis* ATCC 15697]	853 (9)	43
9	gi|23465372	choloylglycine hydrolase [*B. longum* NCC2705]	852 (9)	44
10	gi|490332590	choloylglycine hydrolase [*B. pseudocatenulatum*]	705 (7)	37
11.1	gi|551237101	choloylglycine hydrolase [*B. pseudolongum*]	518 (6)	25
11.2	gi|551237101	choloylglycine hydrolase [*B. pseudolongum*]	503 (6)	25
11.3	gi|551237101	choloylglycine hydrolase [*B. pseudolongum*]	664 (7)	30
12	gi|296454100	choloylglycine hydrolase [*B. longum* subsp. *longum* JDM301]	899 (8)	39

Protein samples were cut out of all BSH-active bands from native polyacrylamide gel (Figure 3). Sample origin: 1, *B. adolescentis* DSM 20087; 2, *B. animalis* subps. *animalis* NRRL B-41406; 3, *B. animalis* subps. *lactis* NRRL B-41405; 4.1, 4.2; *B. bifidum* DSM 20456; 5, *B. breve* DSM 20091; 6, *B. breve* NRRL B-41408; 7, *B. catenulatum* DSM 20224; 8, *B. infantis* ATCC 15697; 9, *B. longum* NRRL B-41409; 10, *B. pseudocatenulatum* DSM 20439; 11.1, 11.2, 11.3; *B. pseudolongum* DSM 20099; 12, *B. suis* NRRL B-41407.

a Protein scores derived from individual ion scores of Mascot-identified tryptic peptides as a non-probabilistic basis for ranking protein hits.

b Total number of tryptic peptides that the Mascot program assigned to a database protein. To compute this number, multiple matches to bifidobacterial peptides with the same primary sequence but representing different charge or modification states were counted as one.

### Purification and molecular weight estimation of BSH

Preliminary studies examined different techniques of protein purification such as gel filtration (Superose 12 and Sephacryl S300), hydrophobic interaction chromatography (Butyl Sepharose and Phenyl Sepharose) and ion exchange chromatography (Q Sepharose and CM Sepharose). Based on results obtained a two-step chromatographic procedure was developed for chromatographic purification of two bile salt hydrolases from *B. pseudocatenulatum* and *B. longum* subsp. *suis*. Briefly, the cell-free extracts (5 ml) were loaded onto a Butyl-Sepharose hydrophobic interaction chromatography (HIC) column (1×5cm), equilibrated with a 50 mM sodium-phosphate buffer containing 0.8 M sodium sulfate. The bound enzymes were eluted by a 50 mM sodium-phosphate buffer with a decreasing gradient of sodium sulfate (0.8 – 0 M) at a flow rate of 1 ml/min. Fractions exhibiting bile salt hydrolase activity were pooled, desalted and concentrated using a Vivaspin 20 centrifugal concentrator (10,000 MWCO, Sartorius). The concentrated enzyme solutions were applied onto a Q-sepharose anion-exchange chromatography column (1×10 cm), that was equilibrated with a 50 mM bis-Tris propane buffer (pH 6.5). The enzyme was eluted at a flow rate of 1 ml/min by using a linear gradient of 0–1 M sodium chloride in 50 mM bis-Tris propane buffer (pH 6.5). The active fractions were then concentrated and used for further characterization. To determine the homogeneity and the accurate subunit molecular weight of the enzymes, protein samples were separated via 12% sodium dodecyl sulfate-polyacrylamide gel electrophoresis according to Laemmli [28]. The protein bands were visualized by staining with Coomassie Brilliant Blue R-250. The native molecular mass of BSH was estimated by the size exclusion chromatography (Sephacryl S-300 HR, Amersham Pharmacia Biotech) using the molecular weight standard kit obtained from Biorad. The column (1.5×100 cm) was pre-equilibrated with a 0.1 M sodium-phosphate buffer (pH 7.0) containing 0.15 M sodium chloride. The enzyme was eluted by using the same buffer at a flow rate of 0.5 ml/min. All chromatographic separations were performed using a BioLogic Duo Flow system (Biorad).

### Isoelectric focusing

For isoelectric focusing of purified bile salt hydrolases protein samples (100 µl) were prepared with a 2D Clean-Up kit (Amersham Biosciences) and then protein pellets were dissolved in a rehydration buffer (Bio-Rad) containing 8M urea, 2% (w/v) CHAPS, 50 mM DTT, 0.2% (v/v) Bio-Lyte 3/10 ampholyte, and 0.001% (w/v) Bromophenol Blue. Proteins were subjected to active rehydration (12 h; 50 V; 20°C) on 7 cm, pH 3–6, linear IPG strips and then to isoelectric focusing by using Protean IEF (Bio-Rad) for a total of 10 kVh at 20°C under mineral oil to prevent evaporation. The proteins were visualized by Coomassie Brilliant Blue R-250 staining.

### Partial characterization of purified bile salt hydrolases

To determine the temperature optima of purified enzymes, BSH activity was measured by incubating standard reaction mixtures (200 µl) at different temperatures ranging from 25 to 70°C. The activity was also examined at different pH values using a 50 mM acetate buffer (pH 4–5.6) and a 0.1 M phosphate buffer (pH 5.7–8.0). The sensitivity of the BSHs to metals and inhibitors was tested using a 0.1 M phosphate buffer pH 6.0 containing 10 mM 2-mercaptoethanol and appropriately diluted protein sample. Because some chemicals interact with phosphate buffer, 20 mM MES buffer (pH 6) was used in some cases. Assays were performed using the following compounds: *p*CMBA, iodoacetamide, *N*-ethylmaleimide, periodic acid, HgCl_2_, CuCl_2_, CaCl_2_, MgSO_4_, and NaCl (final concentrations of all tested chemicals are presented in Table 3). All mixtures were incubated at 37°C for 30 min. Reactions were conducted under standard conditions described above, with 10 mM TDCA as the substrate. The BSH activity assayed in the absence of metals and inhibitors was recorded as 100%. Substrate specificity was determined using six bile salts (taurocholic acid, taurodeoxycholic acid, taurochenodeoxycholic acid, glycocholic acid, glycodeoxycholic acid, and glycochenodeoxycholic acid) manufactured by Sigma Aldrich.

**Table 3:** Effect of various chemicals on BSH activity.

Reagents	Concentrations (mM)	% Residual activity	
		*B. longum* subsp. *suis*	*B. pseudocatenulatum*
*p*CMBA	0,01	1,7	0,4
iodoacetamide	30	56,4	35,5
*N*-Ethylmaleimide	5	31,4	48,3
periodic acid	3	0	0
HgCl_2_	0.2	2,1	4,1
CuCl_2_	5	0	0
CaCl_2_	30	121,5	121,4
MgSO_4_	30	105,8	103,1
NaCl	50	112,1	110,6

### Rheometry

Rheological measurements were conducted using a Haake RS 300 rheometer (Haake, Karlsruhe, Germany). Temperature control was maintained by a Haake DC30 circulatory water bath (Haake, Karlsruhe, Germany). All rheological data were collected and calculated by Haake Rheowin software version 3.61.0004 (Haake). Rheological measurements were carried out using a concentric-cylinder-fixed cup (21.700 mm radius) and a rotating bob (20.710 mm radius, 55 mm lenght, 3 mm clearance to bottom). Measurement began when the sample (10 ml) was poured into the cup, the lift moved and the bob took the measuring position. Strain sweeps were conducted at a frequency of 1.0 Hz. Dynamic measurements were conducted at the strain corresponding to the maximum found within the linear viscoelastic region of the studied material [29].

For rheological analysis of gelation process of deoxycholic acid under different pH, 10 mM solutions of DCA were prepared in 0.1 M sodium phosphate buffer (pH 6–7). In turn, 0.1 M sodium phospate buffer pH 6.25 was used to prepare samples containing various concentations of DCA. All measurements were conducted at 37°C for 15 min. In the case of observation of hydrogel-forming abilities of bifidobacteria in the presence of TDCA, bacterial strains were grown in a Garche's medium (10 ml) for 24 h, and then cells were harvested by centrifugation at 7,000 ×g for 10 min. The cell pellet was washed twice in a 0.1 M sodium-phosphate buffer (pH 6.25) and resuspended in 10 ml of the same buffer containing 10 mM TDCA. Next, the samples were analyzed using rheometer at 37°C for 5 h.

### Statistical analysis

The data were analyzed by the Microsoft Excel 2007 (Microsoft Corporation) and the Statistical Analysis System (SAS Enterprise Guide 3.0.3.414) using ANOVA procedure for analysis of variance and Tukey's test for ranking the means.

## Results

### Detection of BSH activity

**Figure 1:**
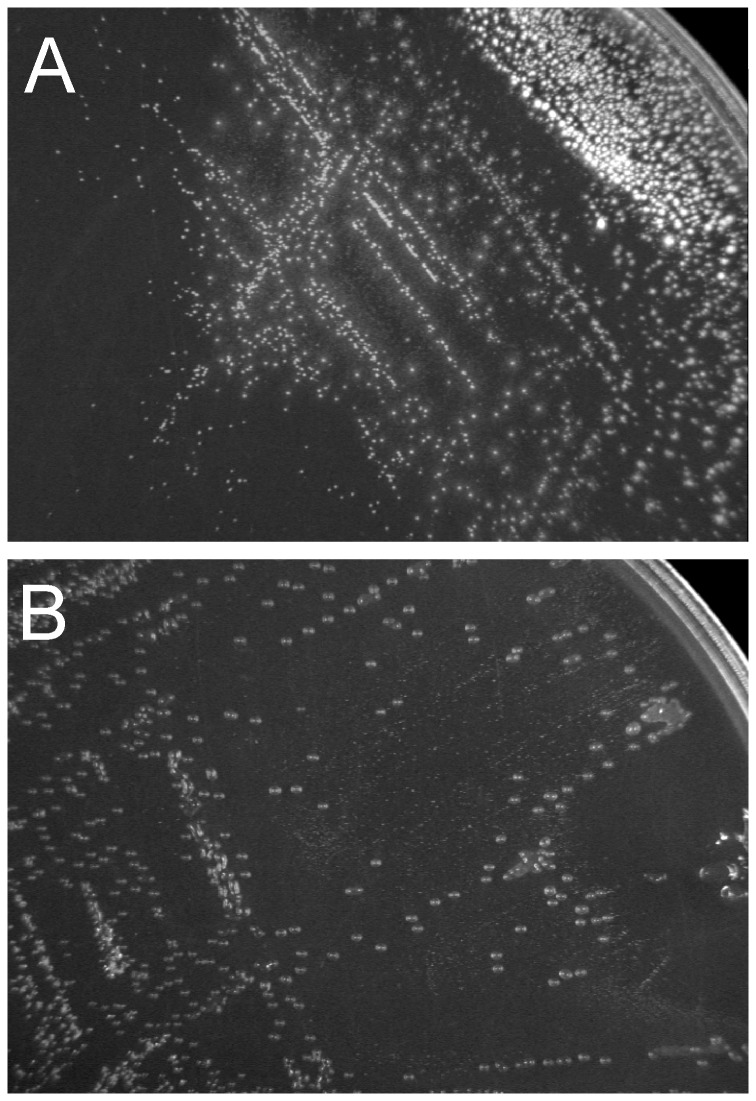
Detection of bile salt hydrolase in *Bifidobacterium* using the plate test. Analysis of colony morphologies of *Bifidobacterium* was performed on solidified MRS (A) and Garche's medium (B) containing bile salts on the example of *Bifidobacterium bifidum* DSM 20456.

In this work, we analyzed the ability of fourteen *Bifidobacterium* strains to deconjugate bile salts. In preliminary experiments we observed the growth of all bifidobacteria tested on solid MRS medium containing sodium taurodeoxycholate (TDCA). In the culture conditions used in this study, twelve strains generated colonies which are characteristic of bile salt hydrolase-active microorganisms (Figure 1A). Of those tested, *B. catenulatum*, *B. longum* subsp. *longum* and *B. longum* subp. *suis* produced opaque white colonies. The deconjugation activity of other BSH-positive bifidobacteria (Table 1) was manifested by the formation of characteristic precipitate halos around the colonies. Interestingly, single colonies of BSH-positive bifidobacteria grown on solidified Garche's medium (containing 0.25 – 0.5% TDCA) were translucent, smooth, circular and additionally viscous (Figure 1B). In both cases (on MRS and Garche's medium), three BSH-negative reference strains (*B. asteroides*, *B. coryneforme* and *Lb. rhamnosus*) produced similar or identical types of colonies on plates with and without TDCA supplementation.

**Figure 2:**
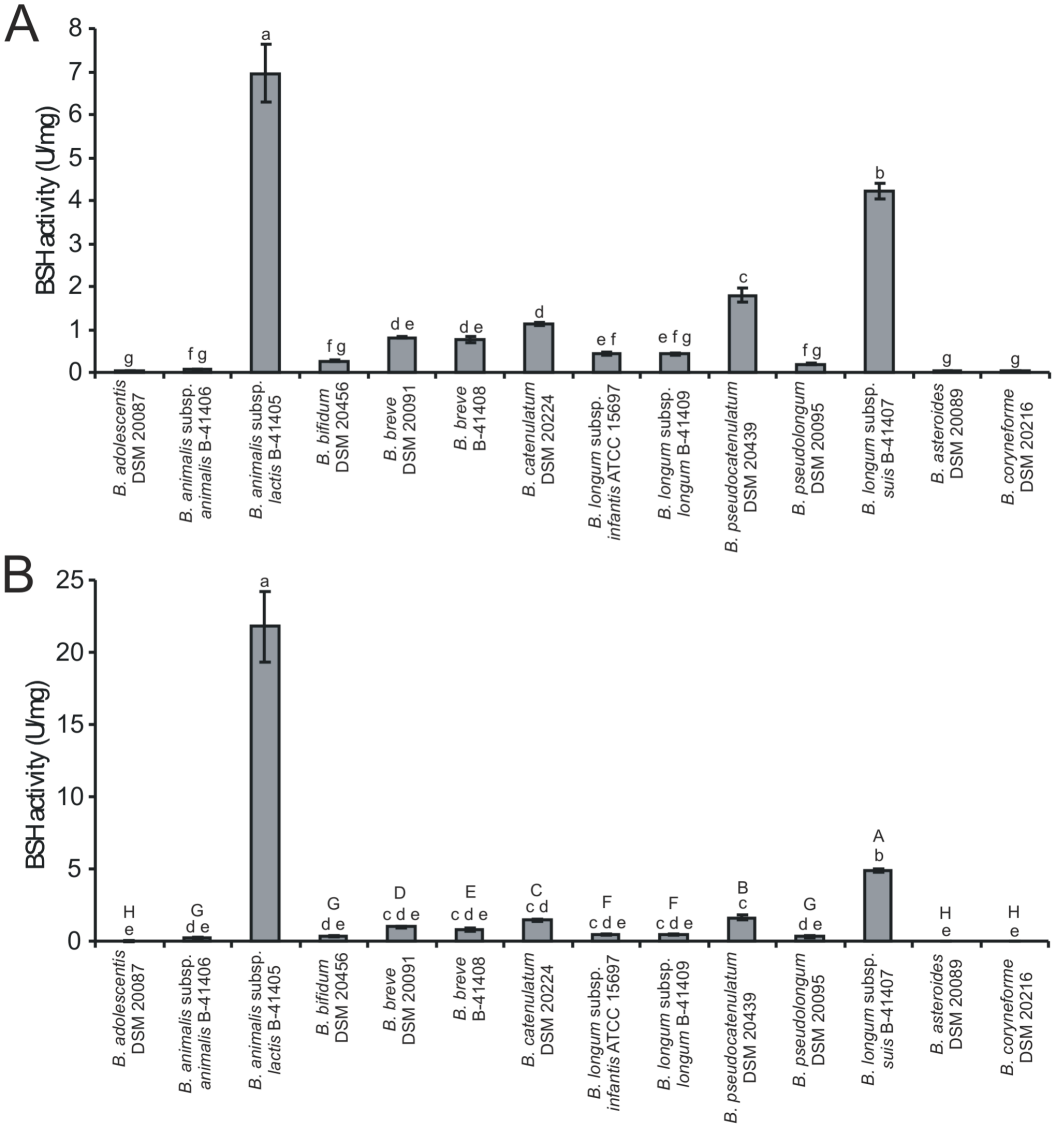
Comparison of bile salt hydrolase activity in fourteen strains belonging to the genus *Bifidobacterium*. BSH activity (units mg^−1^ protein) was measured by determining the amount of amino acids liberated from sodium taurodeoxycholate (A) and sodium glycodeoxycholate (B). Data are presented as the mean of six independent replicates. Error bars represent standard deviation. Different lower case letters designate the means with statistically significant differences (p<0.05). For assays with GDCA (B) capital letters indicate statistically significant differences (p<0.05), excluding results obtained for *B. animalis* subsp. *lactis*.

In the next step of the study, the cell-free extracts obtained from all analyzed bifidobacteria were tested for BSH activity in a two-step procedure previously described by Tanaka et al. [4]. This experiment showed that twelve BSH-positive strains used in this study displayed a different level of deconjugation activity of TDCA and GDCA (Figure 2). As expected, two BSH-negative reference strains (*B. asteroides* and *B. coryneforme*) were negative in the performed assay. Among all bifidobacteria examined, *B. animalis* subsp. *lactis* exhibited definitely the highest specific activity against TDCA (even in comparison with *B. animalis* subsp. *animalis*). The high BSH activity of this strain was also confirmed in enzymatic assays with whole cell suspensions. Such a high deconjugation activity of the probiotic strain, which is commonly used as a food additive seems to be particularly noteworthy, as some researchers suggest that BSH activity may exert detrimental effects to human health. The results also showed high BSH activity for *B. longum* subsp. *suis* and *B. pseudocatenulatum*, while in the case of *B. adolescentis* and *B. animalis* subsp. *animalis* deconjugation activity was relatively low but still detectable. Additionally, BSH enzymes exhibited a preference for glycine-conjugated bile salt (GDCA) over taurine-conjugated form (TDCA), in all tested microorganisms. It was particularly evident in the case of *B. animalis* subsp. *animalis* and *B. animalis* subsp. *lactis*, which showed about three-fold higher BSH activity with glycodeoxycholic acid compared to taurodeoxycholic acid.

### LC MS/MS analysis of native PAGE profiles of BSHs

**Figure 3:**
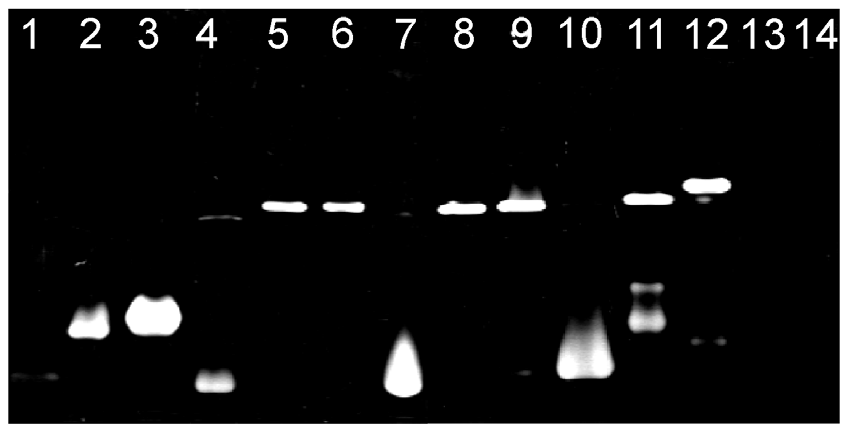
Activity staining on a non-denaturing polyacrylamide gel. Native electrophoresis was performed using 10% nondenaturating acrylamide gel that was stained using reaction buffer containing 10 mM GDCA. The enzymatic activity in the gel was identified by the formation of white precipitate of deoxycholic acid at the position of bile salt hydrolase. Lanes: 1, *B. adolescentis* DSM 20087; 2, *B. animalis* subps. *animalis* NRRL B-41406; 3, *B. animalis* subps. *lactis* NRRL B-41405; 4, *B. bifidum* DSM 20456; 5, *B. breve* DSM 20091; 6, *B. breve* NRRL B-41408; 7, *B. catenulatum* DSM 20224; 8, *B. infantis* ATCC 15697; 9, *B. longum* NRRL B-41409; 10, *B. pseudocatenulatum* DSM 20439; 11; *B. pseudolongum* DSM 20099; 12, *B. suis* NRRL B-41407; 13, *B. asteroides* DSM 20089; 14, *B. coryneforme* DSM 20216.

Previous studies have shown that the analysis of electrophoretic mobility of bile salt hydrolases in nondenaturing conditions may be a useful method for preliminary differentiation of bifidobacteria [6], [30]. In the present work, native polyacrylamide gel electrophoresis of cell-free extracts from fourteen *Bifidobacterium* strains was performed. The resultant gels were incubated in the presence of a 10 mM solution of TDCA or GDCA. The results showed identical activity patterns for both bile salts, with the exception of difficulties in obtaining positive results for bifidobacteria exhibiting low BSH activity when TDCA was used (*B. adolescentis* and *B. animalis* subsp. *animalis*). The results also demonstrated distinct migration patterns of BSH for protein samples of various origins (Figure 3). As in the previous studies, the bile salt hydrolase activity of each tested strain was presented by one, two or three segments with different electrophoretic mobilities [8,31 These results are caused by the complex structure of enzymes and are strongly dependent on the level of BSH activity in the protein samples tested, the quantity of protein sample loaded onto the gel as well as the sensitivity of the BSH activity staining method. Additionally, the analysis of electrophoretic mobility of bile salt hydrolases orginating from twelve bifidobacteria allowed us to differentiate most of the species investigated (Figure 3). Interestingly, in some cases, this method turned out to be a reliable procedure, allowing rapid differentiation of BSH-active bifidobacteria even at the subspecies level.

Subsequently, for definitive identification of BSH enzymes from all tested strains, protein samples were excised from native PAGE gels and afterwards analyzed using mass spectrometry. The obtained MS/MS spectra were evaluated by using the Mascot against NCBI databases. The results allowed unambiguous identification of bile salt hydrolase in all samples examined (Table 2). In most cases, it was also possible to accurately determine the source of the identified enzymes. Due to the high similarity of amino acid sequences of BSH between *B. breve* and *B. longum* (>99%), these two taxa could not be clearly distinguished. Mass spectrometry also revealed that in case of cell-free extracts which more than one BSH active band generated (Figure 3), the same BSH enzyme was detected in all presented segments (Table 2).

**Figure 4:**
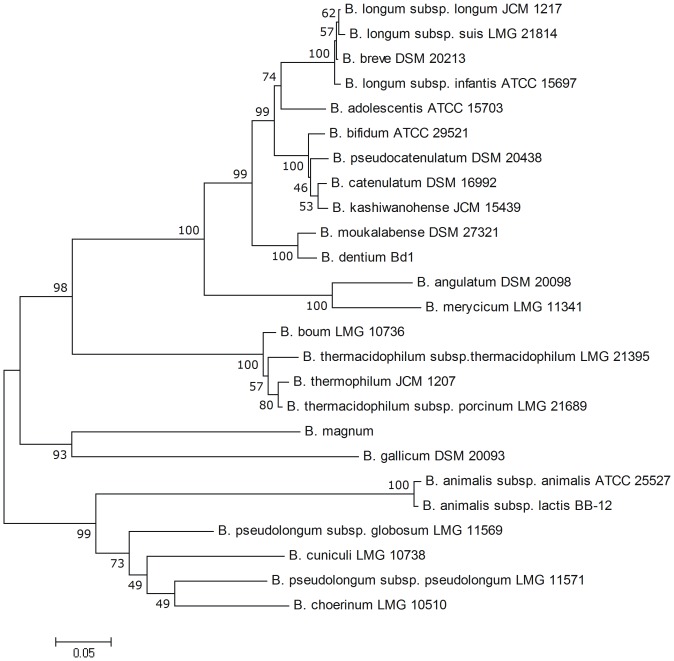
Phylogenetic tree based on amino acid sequence of BSHs from different bifidobacteria. The phylogenetic tree was calculated using neighbor joining method from 1000 bootstrapping replicates with software package MEGA version 4.0. Amino acid sequences used for phylogenetic tree calculation are present in the NCBI database under the following accession numbers: YP_909719.1, KFI89881.1, EFE89535.1, YP_002322914.1, YP_004220577.1, KFI71781.1, AAT11513.1, ETY71513.1, KFI75916.1, EEB21828.1, EDT46256.1, EEP21498.1, KFJ07027.1, KFI79764.1, KFI78707.1, KFI67852.1, EFA23638.1, KFI70893.1, KFI67645.1, KFI47755.1, KFJ00339.1, KFJ02268.1, KFI57613.1, KFI65994.1, YP_006280029.1, YP_002969854.1.

Finally, the phylogenetic analysis of BSH amino acid sequences obtained from NCBI databases was performed. The results showed very high variation of bile salt hydrolases among intestinal bifidobacteria (Figure 4). For the twenty one analyzed species of *Bifidobacterium,* the BSH amino acid sequence similarity varied from 44.4 to 99.7%. These results clearly demonstrate that bile salt hydrolase may be a valuable molecular marker for phylogenetic studies and specific identification of BSH-active *Bifidobacterium* strains.

### Purification and biochemical characterization of bile salt hydrolases from *B. pseudodocatenulatum* and *B. longum* subsp. *suis*

**Figure 5:**
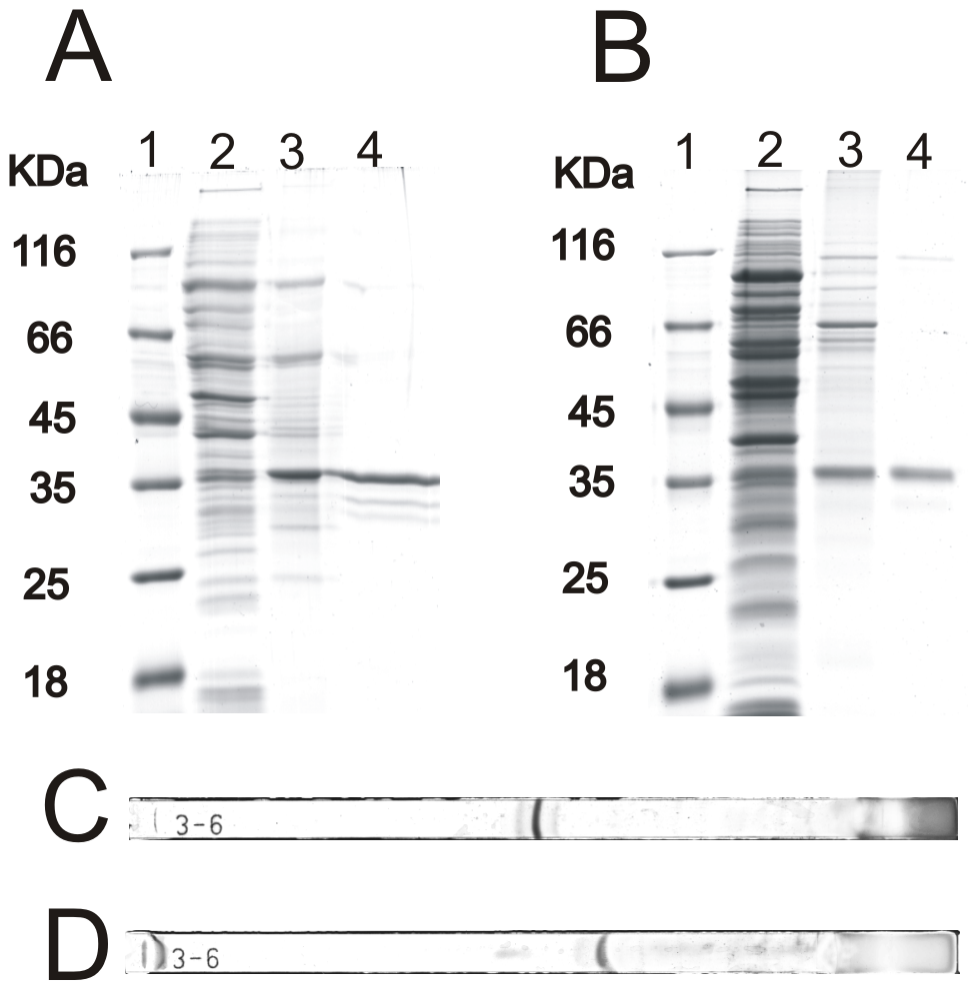
Electrophoretic examination of the tested bile salt hydrolases. SDS-PAGE analysis of fractions obtained during the purification of BSHs from *B. pseudocatenulatum* (A) and *B. suis* (B) was performed on 12% polyacrylamide gels under denaturing conditions. Gels were stained with Coomassie Brilliant Blue R250. Lanes: 1, molecular weight marker; 2, cell-free extract; 3, pooled fractions from hydrophobic interaction chromatography (HIC); 4, active fractions from HIC plus ion-exchange chromatography. Isoelectric point determination of the purified BSHs from *B. pseudocatenulatum* (C) and *B. suis* (D) was performed using 7 cm, pH 3-6, linear IPG strips stained with CBB R250.

Based on high specific deconjugation activity, two bile salt hydrolases (not described previously) from *B. pseudocatenulatum* and *B. longum* subsp. *suis*, were selected for purification and biochemical characterization. Different chromatographic strategies [5], [6], [8], [31] for purification of BSH enzymes were tested in preliminary experiments. A two-step procedure was applied to purify BSHs from selected bifidobacteria. In the first step of purification, each cell-free extract was passed through a hydrophobic interaction column. In both cases, BSH activity was detected in fractions near the end of sodium sulfate gradient (Figure S1A and Figure S2A). Next, desalted and concentrated protein samples were applied to an anion-exchange chromatography column. The proteins exhibiting BSH activity were eluted at sodium chloride concentrations between 0.19 and 0.25 M for *B. suis* (Figure S1B), and between 0.28 and 0.34 M for *B. pseudocatenulatum* (Figure S2B). Subsequently, the purity and the subunit molecular weight of the purified enzymes were examined by SDS polyacrylamide gel electrophoresis (Figure 5A and Figure 5B). Both purified extracts contained proteins with BSH activity migrating as a major band of molecular weight close to 35 kDa. The native weight of the purified enzymes was estimated by gel filtration chromatography at about 107–124 kDa for putative BSH from *B. suis* and at about 123–154 kDa for *B. pseudocatenulatum*. For further identification of putative bile salt hydrolases, protein samples obtained from SDS-PAGE gels were analyzed by liquid chromatography coupled with mass spectrometry. MS/MS spectra obtained allowed unambiguous identification of the analyzed proteins as bile salt hydrolases of *B. longum* (15 unique tryptic peptides with 76% sequence coverage and the Mascot score value of 1687) and *B. pseudocatenulatum* (15 unique tryptic peptides with 60% sequence coverage and the Mascot score value of 7719).

The approximate isoelectric point values for the purified BSHs were also determined. By using an IEF system equipped with a broad pH range strip (pH 3.0 to 6.0, 7 cm), the pI value for bile salt hydrolase from *B. pseudocatenulatum* was estimated to be about 4.45 (Figure 5C). Under the same conditions of separation, the BSH from *B. longum* subsp. *suis* had pI of about 4.7 (Figure 5D).

**Figure 6:**
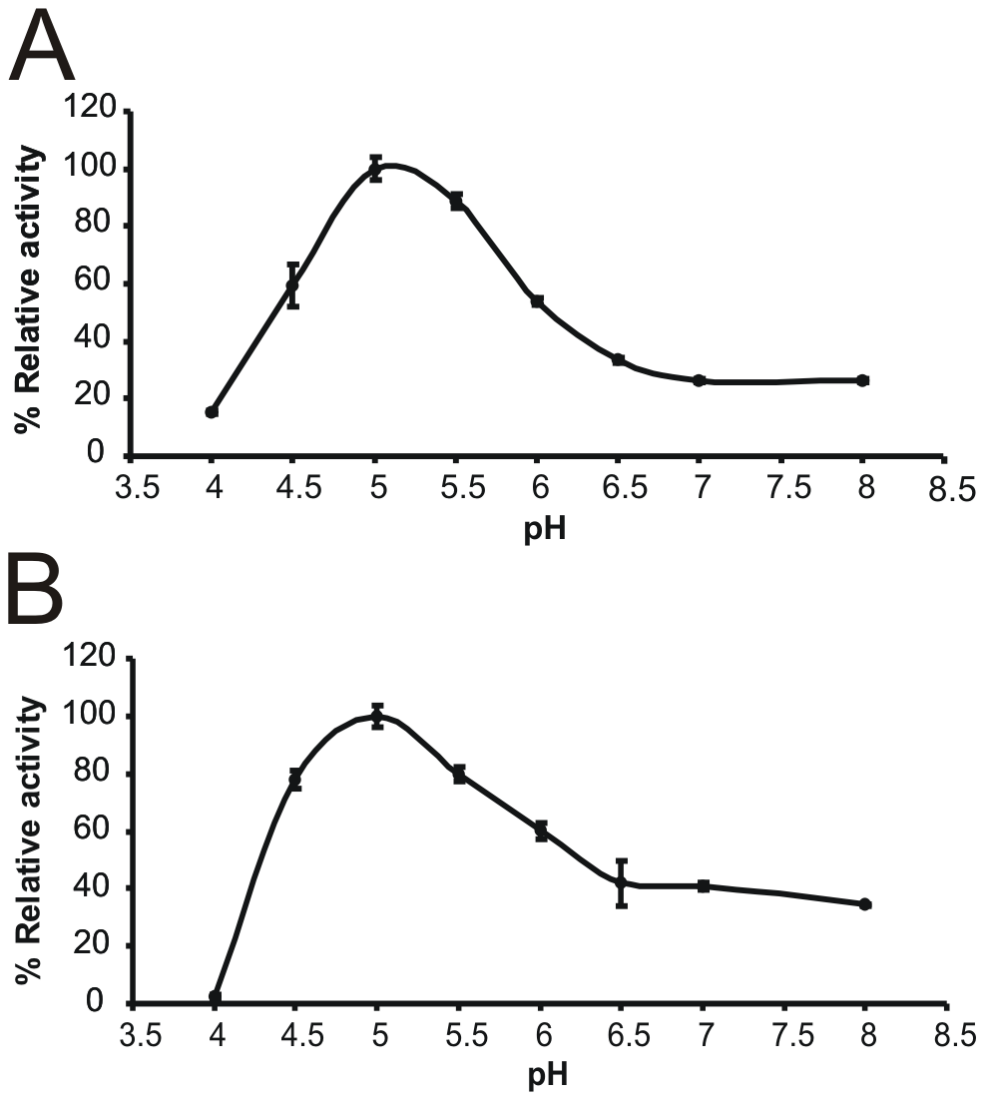
Effect of pH on the activity of the purified bile salt hydrolase from *B. pseudocatenulatum* (A) and *B. suis* (B). Relative deconjugation activity at various pH was calculated using results obtained for pH 5 as a standard at 100%. Values are expressed as the means of three independent replicates. Error bars represent standard deviation.

**Figure 7:**
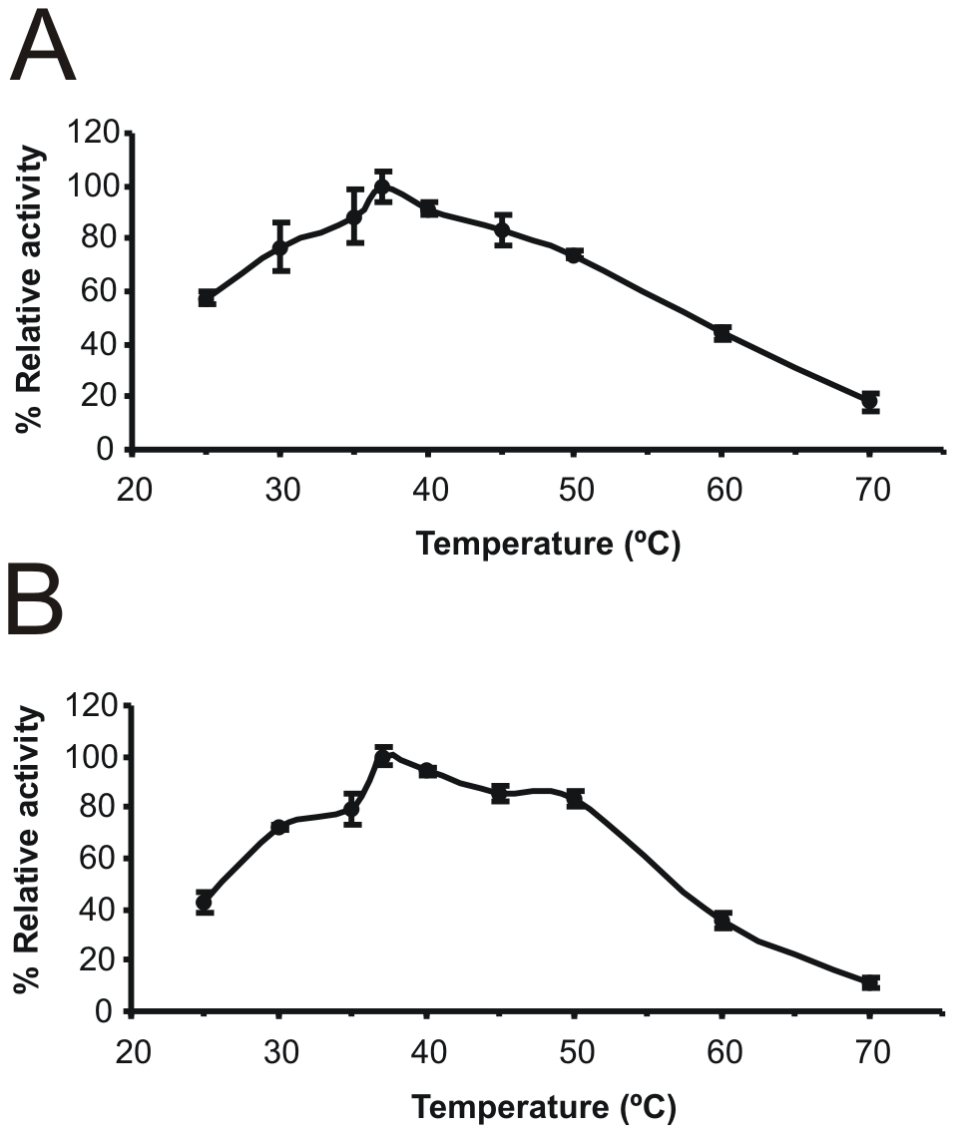
Effect of temperature on the activity of the purified bile salt hydrolase from *B. pseudocatenulatum* (A) and *B. suis* (B). Relative deconjugation activity at various tempearatures was calculated using results obtained at 37°C as a standard at 100%. The results are expressed as the means of three independent replicates. Error bars represent standard deviation.

In the next stage of the research, partial biochemical characterization of purified enzymes was performed. Enzymatic assays showed that both bile salt hydrolases had broad pH optima ranging from pH 4.5 to 6, with the highest activity at pH around 5 (Figure 6). The optimum temperature for bile salt deconjugation activity of the analyzed BSHs was between 30°C and 45°C (maximum at 37°C). A significant decrease of catalytic activity was observed at temperatures higher than 60°C, with 11% and 18% of maximum activity at 70°C for BSH from *B. suis* and *B. pseudocatenulatum*, respectively (Figure 7).

**Figure 8:**
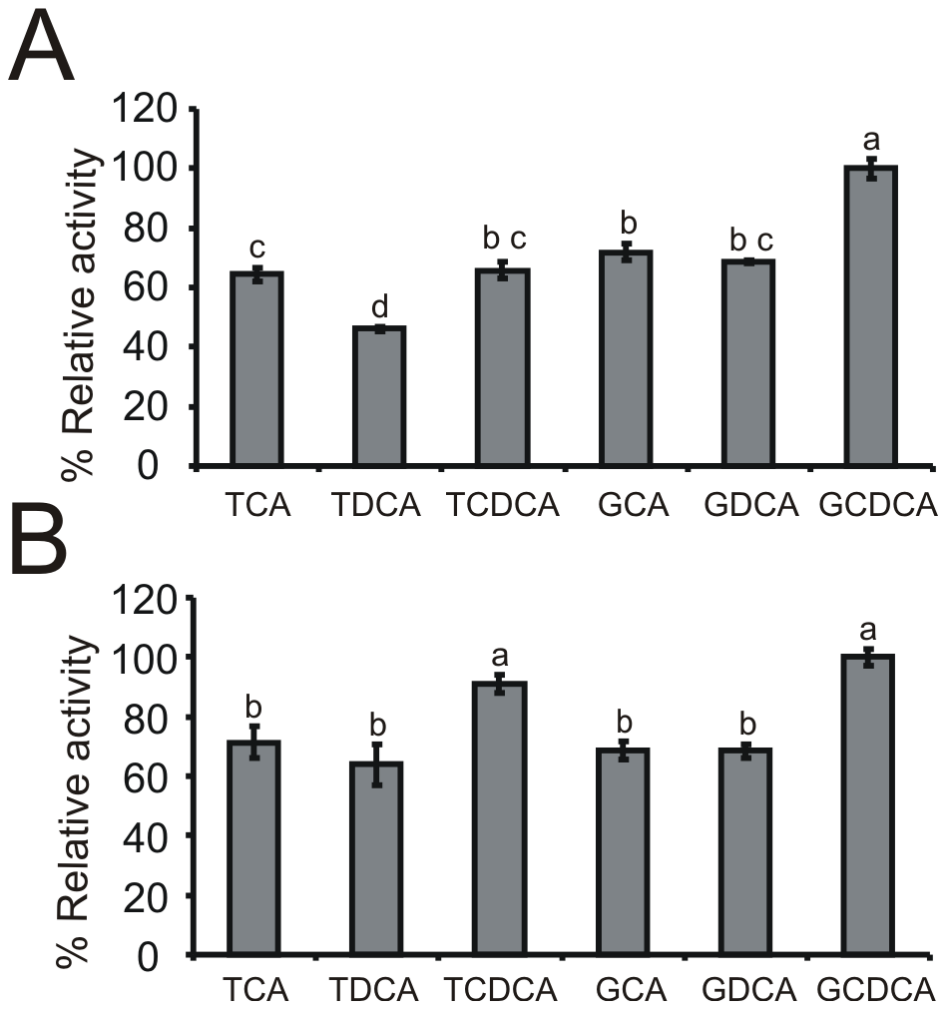
Substrate specificity of the purified BSH from *B. suis* (A) and *B. pseudocatenulatum* (B). Six major bile salts are shown: taurocholic acid (TCA), taurodeoxycholic acid (TDCA), taurochenodeoxycholic (TCDCA), glycocholic acid (GCA), glycodeoxycholic acid (GDCA) and glycochenodeoxycholic acid (GCDCA). Relative deconjugation activity in the presence of six major human bile salts was calculated using GCDCA as a standard at 100%. Values are expressed as the mean of three independent replicates. Different lower case letters indicate statistically significant differences (p<0.05).

The analysis of substrate specificity of BSHs was performed using six major human bile salts (Figure 8). In both cases, the highest enzyme activity was found in enzymatic assays with glycochenodeoxycholic acid (defined as 100% activity). For bile salt hydrolase from *B.suis* clear preference was observed for glycine-conjugated bile salts over taurine-conjugated forms (Figure 8A). This trend was not as evident in the case of BSH from *B. pseudocatenulatum* (Figure 8B). For both analyzed enzymes only a little difference in BSH activity was observed for di- and trihydroxyconjugated bile salts.

The effect of various ions and inhibitors on deconjugation activity of the two purified enzymes was also studied (Table 3). The results showed that among all tested chemicals, the highest inhibition of the analyzed enzymes was obtained in the presence of *para*-chloromercuribenzoic acid (*p*CMBA), periodic acid, HgCl_2_ and CuCl_2_. These substances are generally known as SH enzyme inhibitors, and therefore strongly inhibited the tested BSH enzymes which require a free thiol group for catalytic activity. A slightly lower level of inhibition was also observed with iodoacetamide and *N*-ethylmaleimide.

### Analysis of hydrogel-forming abilities of BSH-positive bifidobacteria in the presence of different bile salts

The analysis of BSH activity performed at our laboratory revealed that the observed enzymatic activity was connected with the production of hydrogel from deconjugated bile acids. This observation is in good agreement with the previously described phenomenon that cholesteric derivatives can form gels in aqueous solutions [33], [34]. Our preliminary experiments, which were conducted using cell free-extracts from different BSH-positive *Bifidobacterium*, the purified enzyme and culture medium containing bile salts showed the formation of hydrogel in all cases. In all experiments we performed a negative control where bile salt hydrolase was absent and in these trials no hydrogel formation was observed. Hydrogel-forming abilities of BSH-positive bifidobacteria were also observed using solidified Garche's plates containing different concentrations (from 0.1 to 1.0% m/v) of taurodeoxycholate and deoxycholate. After 48-h incubation at 37°C, all tested strains which exhibited BSH activity formed translucent and viscous colonies. Such morphology of colonies was obtained on plates containing more than 0.25% taurodeoxycholate, so it was strongly depended on the bile salt concentration (Figure S3). This phenomenon was not observed on the plates supplemented with deoxycholate. Gel-forming colonies were also present on Garche's medium containing another dihydroxyconjugated bile salt - glycochenodeoxycholic acid. Under these experimental conditions translucent and viscous colonies were not observed on the medium supplemented with taurocholic acid (Figure S4).

Additionally, the results obtained in this experiment showed that the analyzed bifidobacteria strains grew definitely better on the medium supplemented with taurodeoxycholate than on plates with deoxycholate. It was especially evident for plates containing more than 0.5% of bile salts. Such results confirm previous reports which suggest that the unconjugated bile acids have a greater inhibitory effect on bacteria than their conjugated forms.

**Figure 9:**
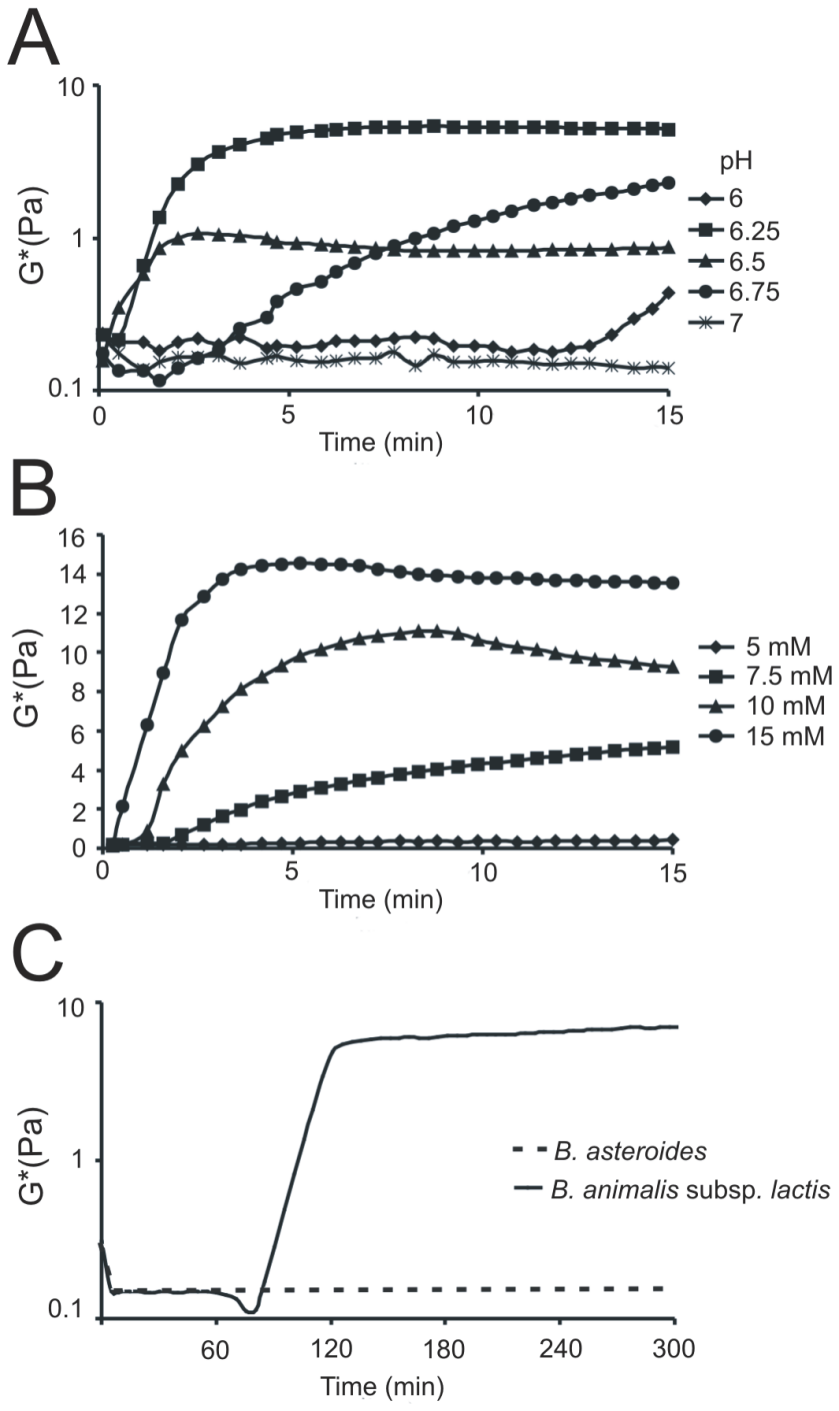
Rheological analysis of the gelation process of deoxycholic acid under different conditions. The effect of various pH (6–7) on viscoelastic behavior of the samples containing 10 mM deoxycholate (A). Analysis of gel formation in the samples containing various concentrations of DCA (from 5 to 15 mM) (B). The bservation of hydrogel-forming abilities of *B. animalis* subsp. *lactis* and *B. asteroides* (BSH-negative reference strain) in the presence of 10 mM TDCA (C). At least three replicates of each experiment were performed with little variation only one example is displayed.

Next, the dynamic rheometry was applied to monitor the gelation of deoxycholic acid without destruction of a gel network by measuring properties at a low strain. The effects of pH and sodium deoxycholate concentration on gelation process were studied. Changes in complex modulus (G*), which indicates viscoelastic behavior of the sample, are shown in Figure 9. The higher the G* values the stronger the gel. Considering the physiological conditions of the digestive system as well as the physicochemical properties of deoxycholic acid, the gelation process was studied in the pH range of 6.0 – 7.0. The results obtained demonstrated that in the applied experimental conditions the most stable gel was formed at pH 6.25 (Figure 9A). Therefore, 0.1 M phoshate buffer pH 6.25 was used in further experiments. The effect of deoxycholic acid concentration on the gelation process is demonstrated in Figure 9B. An increase in DCA concentration caused a noteworthy increase in complex modulus values. It needs to be emphasized that under experimental conditions the gelation process was not observed for solutions containing the conjugated form of DCA – taurodeoxycholic acid (data not shown). Finally, gel-forming abilities of bifidobacteria exhibiting bile salt hydrolase activity were analyzed. To this end, the overnight culture of *B. animalis* subsp. *lactis* was centrifuged and then resuspended in a 0.1 M phosphate buffer (pH 6.25) with the addition of 10 mM TDCA. Changes in complex modulus connected with deconjugation activity of the tested strain were monitored with a rheometer for five hours. The results obtained are shown in Figure 9C, where it can be seen that the reaction of deconjugation of taurodeoxycholic acid to deoxycholic acid evoked changes in viscoelastic behavior of the sample. Similar curves were gained for two other tested strains – *B. suis* and *B. pseudocatenulatum* (data not shown). Interestingly, for *B. asteroides* which is a BSH-negative reference strain, changes in complex modulus were not observed. These results confirm that BSH activity commonly found among intestinal microbiota increases hydrogel-forming abilities of some bile salts.

## Discussion

Previously described investigations showed that bile salt hydrolase activity has been detected in many bacteria from genera *Lactobacillus* [4], [9], [10], [12], *Bifidobacterium* [4]–[8], *Clostridium* [42], *Bacteroides* [43], *Enterococcus* [44], *Brevibacillus* [45], and *Listeria* [40], which were isolated from different environments. Interestingly, metagenomic study described by Jones et al. [41] clearly demonstrated distinct differences between the BSH complement of microorganisms derived from various sources. This phenomenon, which is presumably a consequence of the different composition of bile in various mammalian species, suggests coevolutionary nature of the relationships between the host and the microbiome [41].

It is scientifically documented that among microorganisms considered as probiotics, bifidobacteria display particularly high BSH activity. Tanaka et al. [4] showed that BSH activity was most commonly found in strains isolated from the environment reach in bile salts. However, in the case of the genus *Bifidobacterium*, many isolates originating from other sources, such as various milk products and starter cultures, also exhibited high deconjugation activity against bile salts. Although BSH activity is very common among the intestinal bifidobacteria, only a few *Bifidobacterium* BSHs have been precisely described [5]–[8]. A thorough analysis of these enzymes seems to be especially important, because some researchers suggest that BSH activity, which occurs frequently among bacteria commonly used as food additives (e.g. incorporated into dairy products), may also have detrimental effects on human health [2], [3].

In this study, fourteen strains of bifidobacteria were screened for BSH activity using a plate test, two-step enzymatic reaction and also activity staining on a native polyacrylamide gel. In assays performed, all intestinal strains showed the ability to deconjugate bile salts. It is noteworthy, that *B. animalis* subsp. *lactis*, which was isolated from yoghurt, exhibited definitely the highest specific activity among all bifidobacteria tested. Such a high BSH activity of this strain may be connected with oxygen-resistant nature of this bacteria [46], however, further studies including a large number of isolates of this taxon are needed to confirm this hypothesis. The results also showed that two reference strains (*B.asteroides* and *B. coryneforme*) were indeed BSH-negative in the performed experiments. This is consistent with previous studies, which showed that *Bifidobacterium* strains isolated from the hindgut of honeybee did not exhibit deconjugating activity towards bile salts [4], [35]. Interestingly, BSH-positive strains exhibited distinctly different colony morphology on two microbiological media tested. When bifidobacteria were streaked out on MRS supplemented with TDCA, the tested strains produced opaque white colonies with or without precipitated halos, as previously described by Dashkevitcz and Feigher [36]. On Garche's medium, bile salt activity was manifested in the form of translucent and viscous colonies that were unique to BSH-positive strains. In our opinion, the morphology of bile salt hydrolase-active strains on Garche's medium containing TDCA may be very useful for the selective isolation of bacteria exhibiting high BSH activity and may promote BSH as a useful enzymatic marker in certain genetic studies [36], [37].

The analysis of electrophoretic mobility of bile salt hydrolases demonstrated that BSH activity staining on a native polyacrylamide gel may be a valuable method for rapid selection of BSH-positive bifidobacteria [30]. The results showed also the reliability of the LC MS/MS analysis of bile salt hydrolase as a useful procedure for the identification of different *Bifidobacterium* species. Despite its relatively high analysis cost, this procedure seems to be an interesting alternative to genotypic methods, especially when universal primers for *bsh* genes amplification [32] fail to give expected PCR products. Moreover, phylogenetic study of amino acid sequences of BSHs confirmed that bile salt hydrolase is a useful molecular marker for differentiation and specific identification of intestinal bifidobacteria [32].

Similarly to the previous work [8], two bile salt hydrolases from *B. pseudocatenulatum* and *B. suis* were purified by a two-step chromatographic procedure which included hydrophobic interaction chromatography combined with anion-exchange chromatography. The molecular weight of expected monomers for both purified enzymes was estimated using SDS-PAGE to be approximately 35 kDa. In both cases, the native molecular weight was estimated to be significantly above 100 kDa. These results are consistent with previous reports showing that bile salt hydrolases in *Bifidobacterium* strains generally have a complex structure consisting of four identical subunits [5], [6], [8]. Additionally, the pI values were in the range between 4.0 and 5.0, which is also characteristic of the enzymes from the genus *Bifidobacterium* [6], [8].

Biochemical characterization of *B. pseudocatenulatum* and *B. suis* BSHs showed that the purified enzymes hydrolyzed all of the six major human bile salts, preferring more efficient deconjugation of glycoconjugated bile salts than the tauroconjugated bile salts. The obtained data also demonstrated a high activity of the hydrolases under pH and temperature conditions commonly found in the human gastrointestinal tract. Lastly, based on the results from enzyme inhibition studies, the importance of free thiol groups at the bile salt hydrolase active center was also confirmed [5], [8], [31].

It is well documented that some cholesteric derivatives display aqueous media gelating properties [38], [39]. It also concerns the bile acids that occur naturally in the human gastrointestinal tract. Although several authors showed that the gelation process is not a common characteristic of all bile salts, it has been previously described that monohydroxy and dihydroxy bile salts, which are essential components of the human bile, generally form gels [2], [38]. Analysis of bile acid composition in the gallbladder clearly demonstrates that dihydroxycholic acids and monohydroxycholic acids constitute only 60% of all bile acids in the upper parts of the gastrointestinal tract. In the small intestine, a large part of the bile (about 35%) is composed of cholic acid, which generally does not form a gel. The situation is considerably different in the large intestine, due to the very efficient bile salt transport in the gut. Majority of the primary bile acids in the distal ileum is reabsorbed and only about 5% of initial amount passes into the large intestine, which constitutes a natural ecological niche of *Bifidobacterium*. More than 90% of bile salts in this part of the human gastrointestinal tract are able to form hydrogel [2], [47].

However, until now the gelation phenomenon of bile salts was not connected with BSH activity found among *Bifidobacterium*. The investigations performed in this work demonstrated that the release of deconjugated bile acids led directly to the gelation phenomenon of both enzymatic reaction solutions containing purified BSH and culture medium supplemented with bile salts, after the growth of BSH-positive bifidobacteria. In addition, no gelation effect was observed in the experiments, in which BSH enzyme or bile salts were omitted.

In our opinion, if such a phenomenon occurs in physiological conditions of the human gut it may have a significant impact on many factors, such as adherence, autoaggregation and biofilm formation which are key elements of effective bacterial colonization of the human gastrointestinal tract. Furthermore, preliminary studies have demonstrated that in presence of conjugated bile salts the BSH-positive *Bifidobacterium* showed a considerable decrease in susceptibility to some antibiotics. This was not observed for *B. asteroides* and *Lb. rhamnosus*, which are generally considered BSH-negative bacteria. This may suggest that the occurrence of a natural hydrogel shield may provide a survival advantage for strains with high BSH activity.

## Conclusions

In conclusion, this work presents molecular and biochemical characterization of bile salt hydrolases synthesized by intestinal bacteria from the genus *Bifidobacterium*. Deconjugation activity of twelve analyzed strains was shown using a plate test, two-step enzymatic reaction and also activity staining on a native polyacrylamide gel. Subsequently, two bile salt hydrolases from *B. pseudocatenulatum* and *B. longum* subsp. *suis* were purified and characterized. Biochemical analysis revealed that both tested enzymes were able to hydrolyze all of the six major human bile salts under pH and temperature conditions commonly found in the human intestines. Finally, gel-forming abilities of bifidobacteria exhibiting bile salt hydrolase activity were analyzed by dynamic rheometry. The investigations performed demonstrated that the release of deconjugated bile acids led to the gelation phenomenon of reaction solution containing BSH-active bifidobacteria. The results also suggest a new approach to the physiological role of BSH in which we would like to combine microbiological observations, biochemical properties of analyzed enzymes and finally physicochemical characteristics of bile acids. Because the deconjugated bile acids which are products of BSH activity have intrinsic capability to form hydrogels in aqueous solutions, we therefore propose that bile salt deconjugation may be an important factor in efficient colonization and persistence of BSH-positive strains in intestines. Moreover, we hope that our research will contribute to a better understanding of the complex interactions between intestinal bacteria and the human organism. Furthermore, new information about the BSH activity in bifidobacteria can also lead to a more conscious use of living microorganisms as food additives and the development of new medicines for the prevention and treatment of gastrointestinal disorders.

## Supporting Information

Figure S1 **Chromatographic purification of bile salt hydrolase from** ***B. longum*** **subsp.** ***suis*** **.** (A) Elution profile for BSH from Butyl Sepharose hydrophobic interaction chromatography. Solid lane: Protein (A 214 nm); dotted line: sodium sulfate gradient; dashed line: BSH activity. (B) Elution profile for BSH from Q Sepharose anion-exchange chromatography column. Solid lane: Protein (A 214 nm); dotted line: sodium chloride gradient; dashed line: BSH activity.
(TIF)

Figure S2 **Chromatographic purification of bile salt hydrolase from** ***B. pseudocatenulatum*** **.** (A) Elution profile for BSH from Butyl Sepharose hydrophobic interaction chromatography. Solid lane: Protein (A 214 nm); dotted line: sodium sulfate gradient; dashed line: BSH activity. (B) Elution profile for BSH from Q Sepharose anion-exchange chromatography column. Solid lane: Protein (A 214 nm); dotted line: sodium chloride gradient; dashed line: BSH activity.
(TIF)

Figure S3 **Analysis of colony morphology of** ***Bifidobacterium animalis*** **subsp.** ***lactis*** **on solid Garche's medium containing various concentrations of taurodeoxycholate.** The tested strain was grown on plates without bile salts (A) and with the addition of 0.1% (B), 0.25% (C), 0.5% (D), 0.75% (E), and 1% of TDCA.
(TIF)

Figure S4 **Analysis of colony morphology of** ***Bifidobacterium animalis*** **subsp.** ***lactis*** **on solid Garche's medium containing 0.5% (w/v) of taurocholate (A), taurodeoxycholate (B) and glycochenodeoxycholate (C).**
(TIF)
