# Supplementary figures and images for: A New Insight into the Physiological Role of Bile Salt Hydrolase among Intestinal Bacteria from the Genus Bifidobacterium
Source: PLoS One. 2014 Dec 3;9(12):e114379. doi: 10.1371/journal.pone.0114379 (PMC4255033; doi:10.1371/journal.pone.0114379)

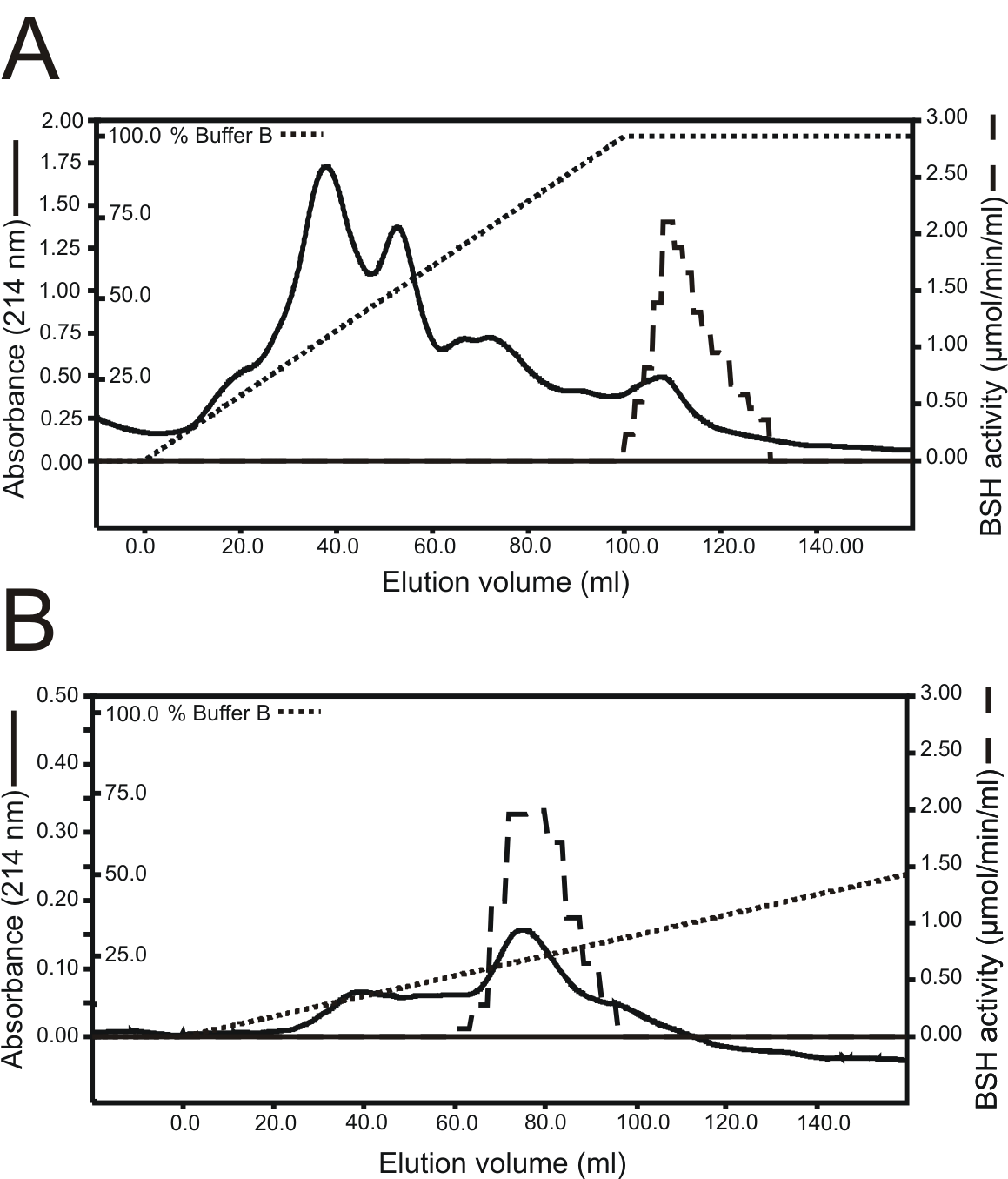

Supplement: Figure S1 — Chromatographic purification of bile salt hydrolase from B. longum subsp. suis . (A) Elution profile for BSH from Butyl Sepharose hydrophobic interaction chromatography. Solid lane: Protein (A 214 nm); dotted line: sodium sulfate gradient; dashed line: BSH activity. (B) Elution profile for BSH from Q Sepharose anion-exchange chromatography column. Solid lane: Protein (A 214 nm); dotted line: sodium chloride gradient; dashed line: BSH activity. (TIF) [file pone.0114379.s001.tif]

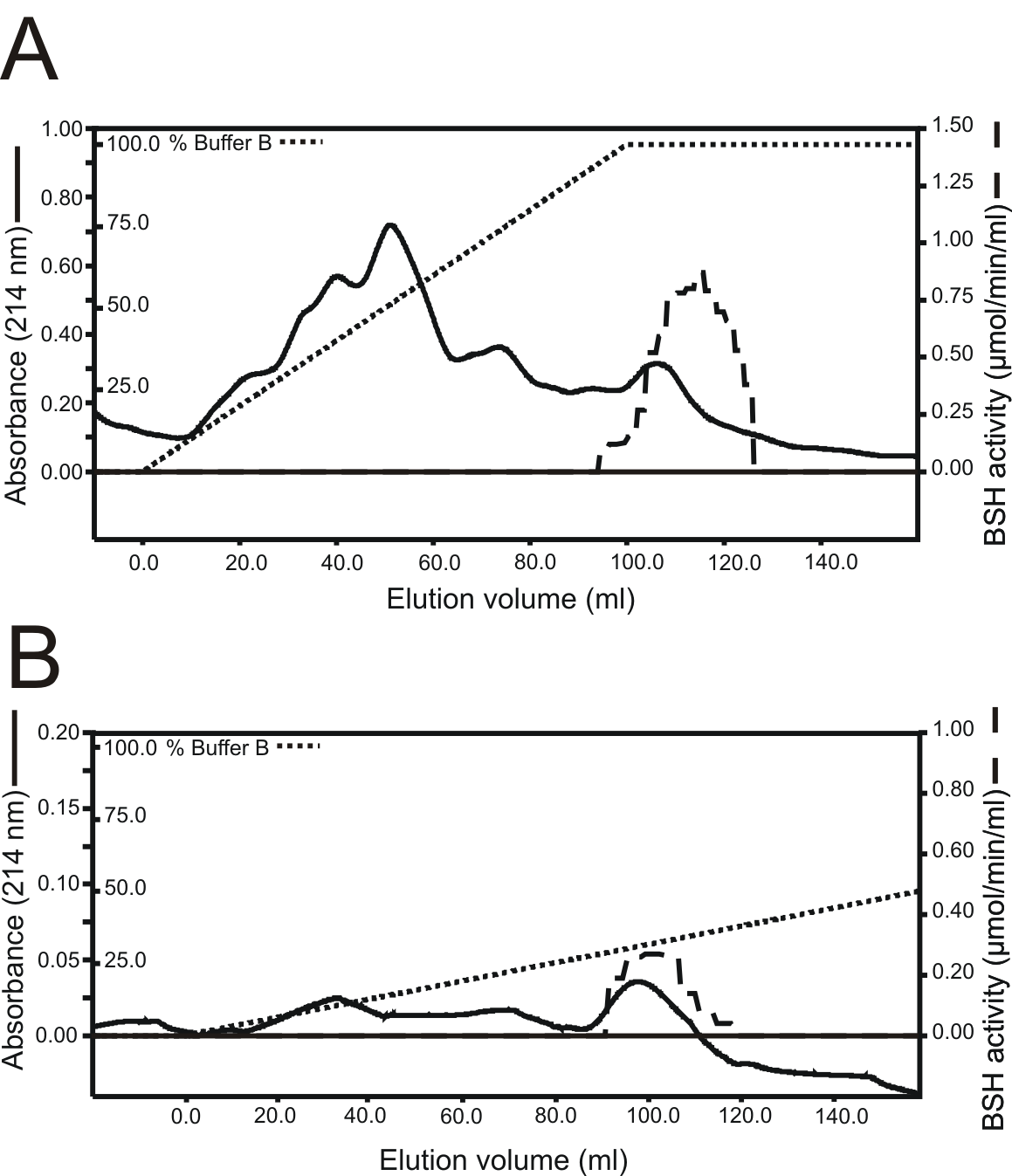

Supplement: Figure S2 — Chromatographic purification of bile salt hydrolase from B. pseudocatenulatum . (A) Elution profile for BSH from Butyl Sepharose hydrophobic interaction chromatography. Solid lane: Protein (A 214 nm); dotted line: sodium sulfate gradient; dashed line: BSH activity. (B) Elution profile for BSH from Q Sepharose anion-exchange chromatography column. Solid lane: Protein (A 214 nm); dotted line: sodium chloride gradient; dashed line: BSH activity. (TIF) [file pone.0114379.s002.tif]

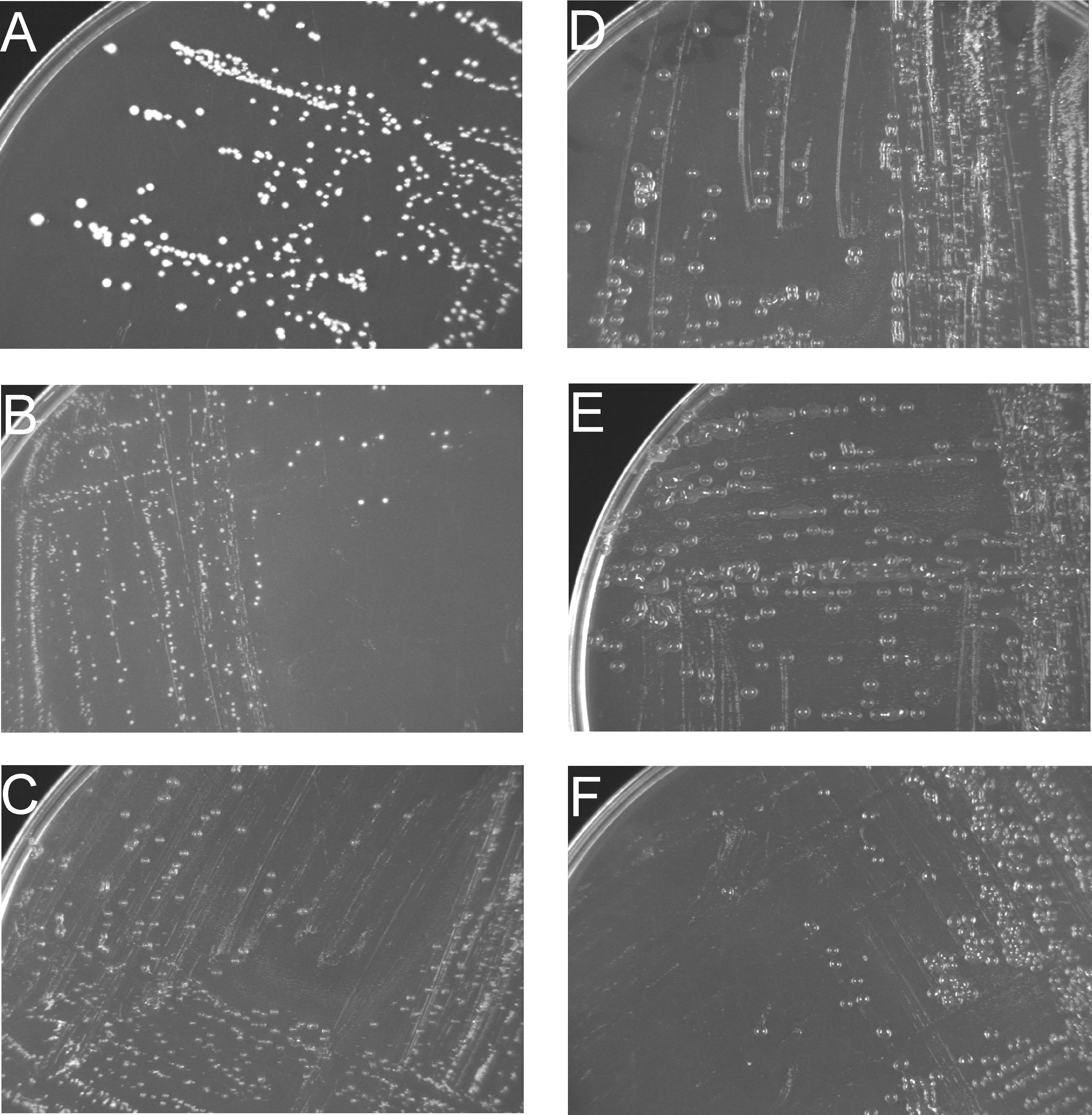

Supplement: Figure S3 — Analysis of colony morphology of Bifidobacterium animalis subsp. lactis on solid Garche's medium containing various concentrations of taurodeoxycholate. The tested strain was grown on plates without bile salts (A) and with the addition of 0.1% (B), 0.25% (C), 0.5% (D), 0.75% (E), and 1% of TDCA. (TIF) [file pone.0114379.s003.tif]

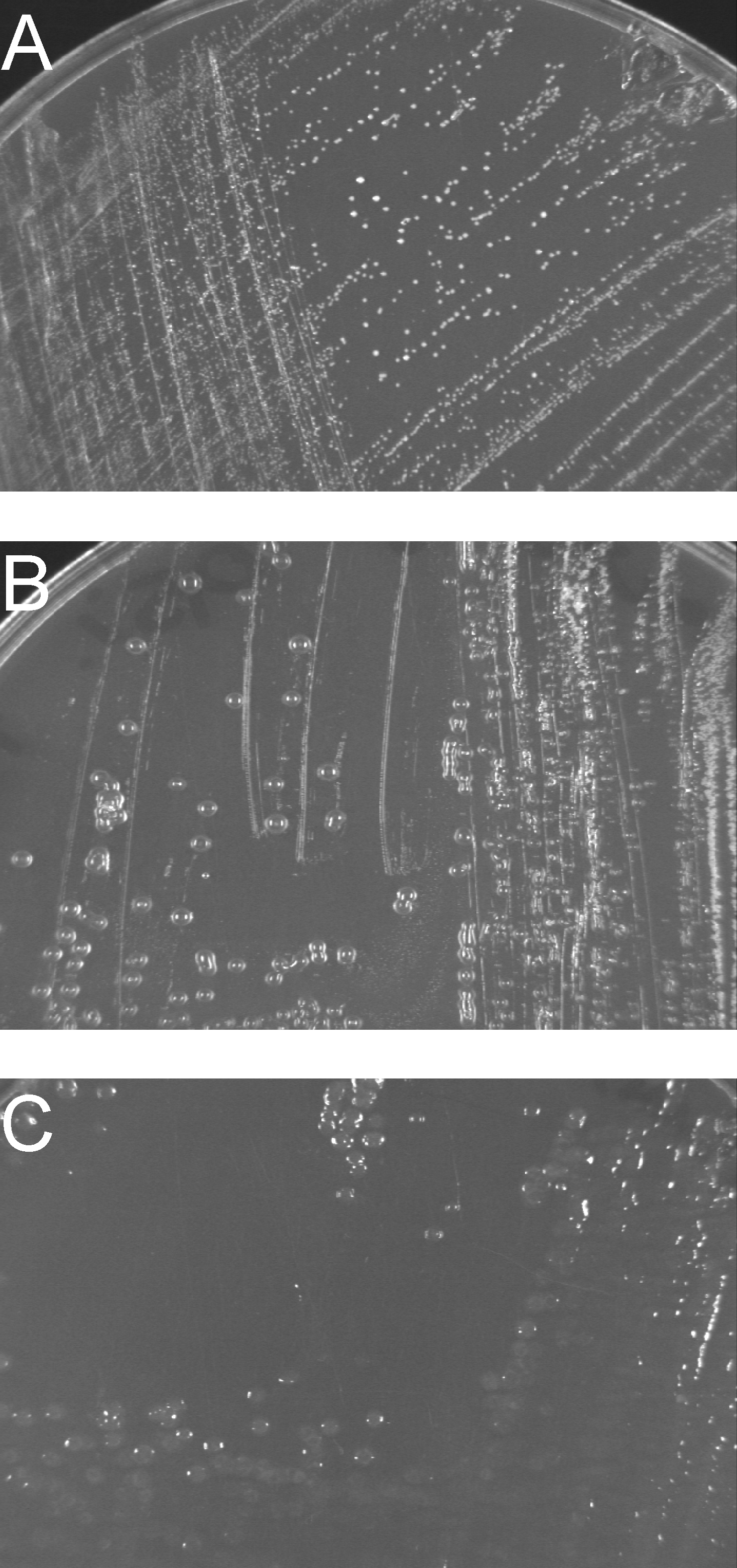

Supplement: Figure S4 — Analysis of colony morphology of Bifidobacterium animalis subsp. lactis on solid Garche's medium containing 0.5% (w/v) of taurocholate (A), taurodeoxycholate (B) and glycochenodeoxycholate (C). (TIF) [file pone.0114379.s004.tif]
